# Cytoskeleton and Membrane Organization at Axon Branches

**DOI:** 10.3389/fcell.2021.707486

**Published:** 2021-09-01

**Authors:** Satish Bodakuntla, Hana Nedozralova, Nirakar Basnet, Naoko Mizuno

**Affiliations:** ^1^Laboratory of Structural Cell Biology, National Heart, Lung, and Blood Institute, National Institutes of Health, Bethesda, MD, United States; ^2^National Institute of Arthritis and Musculoskeletal and Skin Diseases, National Institutes of Health, Bethesda, MD, United States

**Keywords:** cytoskeleton, mitochondria, actin, microtubules, endoplasmic reticulum, axon branching, membraneremodeling and neurons

## Abstract

Axon branching is a critical process ensuring a high degree of interconnectivity for neural network formation. As branching occurs at sites distant from the soma, it is necessary that axons have a local system to dynamically control and regulate axonal growth. This machinery depends on the orchestration of cellular functions such as cytoskeleton, subcellular transport, energy production, protein- and membrane synthesis that are adapted for branch formation. Compared to the axon shaft, branching sites show a distinct and dynamic arrangement of cytoskeleton components, endoplasmic reticulum and mitochondria. This review discusses the regulation of axon branching in the context of cytoskeleton and membrane remodeling.

## Introduction

The brain function depends on a vast number of intricate connections between neurons to process and transmit information. To fulfill the scale and complexity of these connections, it is critical for the neurons to develop properly and maintain neuronal homeostasis over the life span of the organism. To achieve this arduous task, the primary step for neurons is to undergo a polarity establishment process with two main compartments, the somato-dendritic and the axonal compartments, which are distinct in their morphology, molecular composition and subcellular functions. In addition to establishing neuronal polarity, axon outgrowth and branching are vital steps to ensure proper connectivity and development of the brain. While axonal outgrowth is mainly linked to axon guidance and pathfinding (McCormick and Gupton, 2020), branching of axons is crucial for the intertwining of neuronal circuits through synaptic contacts (Kalil and Dent, 2013; Hoersting and Schmucker, 2021). The axonal branching process is balanced by the ability of axons on the one hand to make collateral protrusions establishing synaptic connections but also to retract protrusions eliminating synapses, a process called synaptic pruning (discussed in Gibson and Ma, 2011).

Early studies have shown that the primary growth cone dynamics is linked to the formation of axon branches. Axons do not continuously grow, but rather pause intermittently between growth phases (Harris et al., 1987; Yamamoto et al., 1997). These paused points contain remnants of growth cone components, which act as precursors for axon branching sites (Szebenyi et al., 1998). Axonal branches are formed in two major ways: (1) splitting of the growth cone to create Y- or T-shaped structures with two growing axonal paths, (2) *de novo* branching from the axon shaft, called collateral branch formation. Although both possibilities exist, the latter mechanism of collateral branch formation appears to be the major mechanism (Harris et al., 1987; O’Leary et al., 1990). Understanding axon branching is a challenging task as several pathways have to work together in a highly coordinated fashion on an intracellular as well as intercellular level. Moreover, neuronal cells are extremely sensitive to stress and environmental cues, which impedes many cell biological and biochemical approaches. There are several key questions in axon branching: What determines the position of a branch on the axon shaft? Which mechanisms stabilize axonal branches and allow them to grow further? Which actions are required within a cell as well as on the plasma membrane? How are these actions coordinated in space and time? How do axon guidance cues interconnect with the branching system? Given the paramount importance of axon branching in brain homeostasis, answering these questions is crucial and it will be instrumental for identifying therapeutic targets to alleviate pathological conditions like neurodegenerative diseases. We are only beginning to answer these questions and this review discusses recent advances in obtaining a better picture of the cellular organization of processes governing axon branching with an emphasis on cytoskeleton and membrane trafficking.

## Cytoskeleton Organization and Dynamics at the Axonal Branch

**FIGURE 1:**
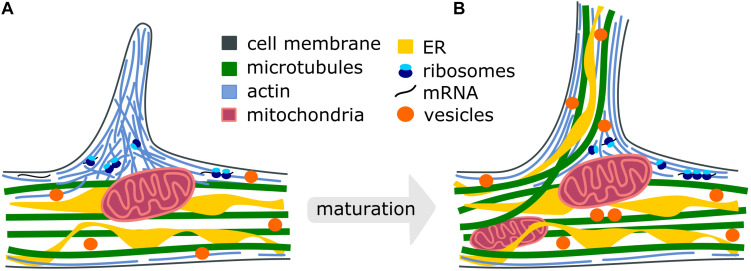
Cellular processes during the maturation of axon branches. **(A)** Premature branch: Actin patches form along the axon and push the plasma membrane to initiate the formation of a filopodium. Mitochondria stall at branch sites and the translation machinery accumulates to locally synthetize new cytoskeletal proteins (Spillane et al., 2013; Armijo-Weingart and Gallo, 2017). **(B)** Mature branch: Dispersed actin filaments reorient and form actin bundles. Microtubules and ER can co-migrate into the growing branch to stabilize it and vesicles supply membrane components. Intracellular organelles such as mitochondria, endoplasmic reticulum, synaptic and dense-core vesicles redistribute and accumulate at the branch site to support the high demand for energy, proteins, lipid- and membrane components.

One of the important early events in the formation of axon branches is the extensive reorganization of cytoskeletal elements (Gallo, 2011). The first observed event during branch formation is the extension of plasma membrane protrusions filled with actin-based components, either finger-like filopodia or sheet-like lamellipodia (Figure 1). The actin-rich protrusions originate from the local accumulation of short pieces of actin filaments along the axonal shaft, called actin patches (Spillane et al., 2011). Several actin-associated signaling proteins like Rho GTPases (Hall, 1998; Hall and Lalli, 2010) and actin remodeling proteins, such as ENA/VASP (enabled/vasodilator-stimulated phosphoprotein) and nucleator complexes like ARP2/3 (actin-related protein 2/3), play important roles in filopodia dynamics resulting in axon development and branch formation (Dwivedy et al., 2007). The activation of Rho in slice cultures of the upper cortical layer results in increased activity-dependent branching (Ohnami et al., 2008). A similar result is observed in cultured hippocampal neurons (Ahnert-Hilger et al., 2004). The reduction of ENA/VASP proteins in the retinal ganglion neurons leads to diminished filopodia formation and axon branching, and the deletion of ARP2/3 in hippocampal neurons reduces the frequency of filopodia (Strasser et al., 2004). ARP2/3 is also required for growth factor-induced branching of sensory axons (Spillane et al., 2011). The duration and frequency of filopodia are shown to affect the number of axonal branches (Sainath et al., 2017). However, while these studies shed light on the importance of the actin cytoskeleton for the initiation of branch formation, their maturation process is not fully understood due to the dynamic nature of actin. The extension of protrusions alone, referred to as premature branches, is not sufficient to stabilize the transformation into a mature axon branch.

The stabilization of transient, premature branches is thought to be mediated by the insertion of axonal microtubules, as the disruption of microtubules causes a reduction in axon branching (Dent and Kalil, 2001). However, the entry of the microtubules into premature protrusions does not necessarily ensure the establishment of a mature branch. It has been shown that longer, microtubule-containing axon branches can regress (Kalil et al., 2000), presumably because microtubules themselves display dynamic instability (Mitchison and Kirschner, 1984; Mitchison, 1993)-switching between phases of microtubule polymerization and depolymerization. Thus, an additional mechanism likely plays a role in facilitating branch maturation (Figure 1). One possibility is the stabilization of microtubules by an interaction with already organized actin filaments in axonal protrusions (Dent and Kalil, 2001; Kalil and Dent, 2014; Sainath and Gallo, 2015). For instance, it has been shown that the actin-binding protein drebrin localizes at branch regions and promotes the entry of microtubules into filopodia, eventually ensuring the formation of mature axon branches (Ketschek et al., 2016). Interestingly, the treatment of neurons with nerve growth factor (NGF) promotes axonal branches (Diamond et al., 1987, 1992; Gallo and Letourneau, 1998; Ketschek and Gallo, 2010; Armijo-Weingart and Gallo, 2017) and also increases the levels of axonal drebrin (Ketschek et al., 2016), suggesting these mechanisms are responsible for changes in extracellular signaling. Septins have also been shown to localize at actin patches during the initiation of axon branching and to regulate the interactions of microtubules and actin in filopodia (Hu et al., 2012). Together, the coordination of microtubule- and actin networks play a crucial role in the formation of axon branches. The relative contribution of these mechanisms to axon branching as well as their coordination in space and time must be further explored.

**FIGURE 2:**
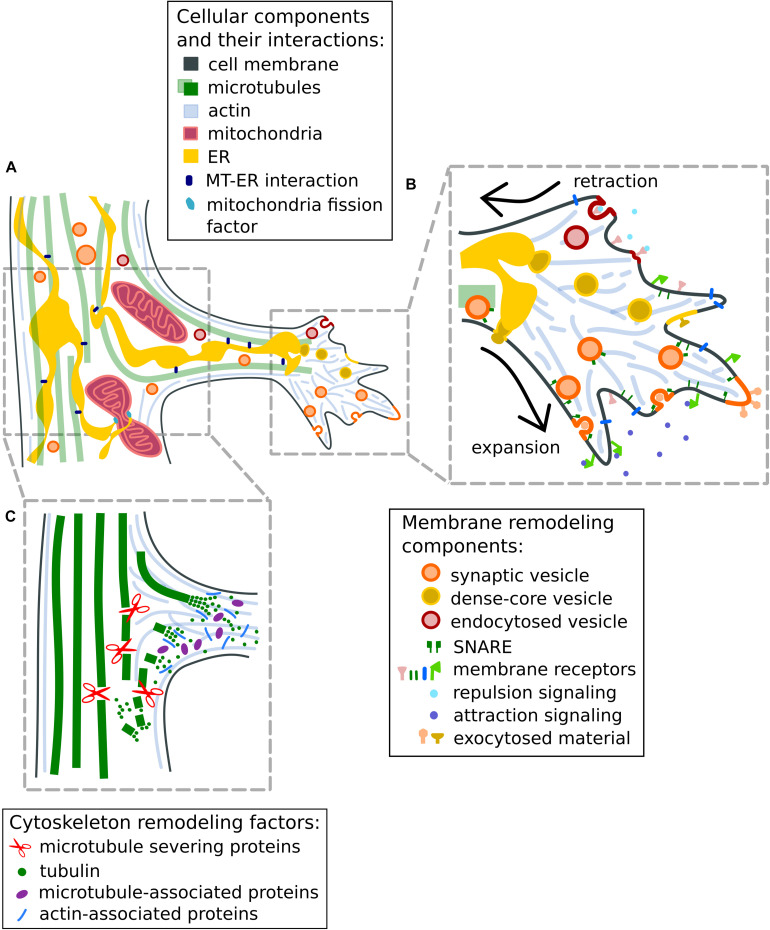
Remodeling of cytoskeleton and plasma membrane at axon branches. **(A)** Schematic representation of a mature, growing axon branch. Mitochondria increase in numbers through fission mediated by mitochondria fission factors or via constriction by the wrapping of endoplasmic reticulum. **(B)** Mechanisms to regulate membrane expansion (1) and retraction (2) at axon branches. Membrane expansion in response to attraction guidance cues is mediated by the fusion of synaptic vesicles with the plasma membrane or by exocytosis using SNARE proteins. The retraction of membranes is initiated by repulsion guidance cues and facilitated by endocytosis of membrane material. **(C)** Mechanisms for the reorganization of the cytoskeleton at axon branches. Bundled microtubule arrays are fragmented by microtubule-severing enzymes such as spastin and katanin to increase the local microtubule/tubulin pool available for polymerization. Tubulin-nucleation factors like MAP7 and SSNA1 promote and stabilize microtubule growth at axonal branches. Actin-microtubule crosslinking factors like drebrins and septins are suggested to promote the entry of microtubules into actin-rich filopodia. Note that processes at the primary growth cone and axon branch formation have similar cellular mechanisms for cytoskeletal rearrangements and responses to attractive or repulsive signaling through exocytosis and endocytosis (discussed in Dent et al., 2003; Winkle et al., 2016).

Moreover, several neuronal microtubule-associated proteins (MAPs) contribute to the maturation of branches by promoting microtubule polymerization or by stabilizing the microtubules at branch sites (Kalil and Dent, 2014; Figure 2). For example, MAP7 (ensconsin or E-MAP-115) promotes microtubule polymerization *in vitro* and it has been shown to accumulate at newly forming axon branches, and to increase the number of axonal branches (Tymanskyj et al., 2017). MAP7 has been shown to enter the branch with a delay and colocalize with stable microtubules, suggesting a specific role in the maturation of branches by microtubule stabilization (Tymanskyj et al., 2017, 2018). Interestingly, MAP7 has been shown to enhance the recruitment of kinesin-1 to microtubules (Tymanskyj et al., 2018; Hooikaas et al., 2019), possibly related to the accumulation of organelles at the axonal branch (discussed below). Similar to MAP7, SSNA1 (Sjoegren syndrome nuclear autoantigen 1, also known as NA14), a microtubule nucleation factor, also accumulates at axon branching sites (Basnet et al., 2018) and its overexpression induces axon branching (Goyal et al., 2014). Interestingly, SSNA1 induces not only microtubule nucleation but also a unique microtubule branching *in vitro* (Basnet et al., 2018).

Early studies have suggested that microtubules undergo destabilization in axons during branch formation, and fragmented microtubules are transported to or generated at newly forming branches (Gallo and Letourneau, 1999; Kalil and Dent, 2014; Armijo-Weingart et al., 2019; Figure 2C), enabling their growth. It appears that microtubule fragmentation at localized areas along the axon shaft would increase the amount of available tubulin molecules or short microtubule fragments for microtubule remodeling, which eventually stabilizes and promotes the formation of mature axon branches (Yu et al., 1994; Kalil et al., 2000). Indeed, overexpression of the microtubule-severing enzymes spastin and katanin increases the localized microtubule mass (Kuo et al., 2019), leading to an increased number of axon branches (Yu et al., 2008). Agreeing with these notions, the destabilization of microtubules by an increase of the local calcium concentration along the axon was also shown to induce new collateral branches (Ziv and Spira, 1997). On the other hand, when exposed to the microtubule-stabilizing drug taxol, neurons show a reduction in the invasion of microtubules into early filopodia and consequently, decreased branch formation (Dent and Kalil, 2001; Dent et al., 2007).

Apart from the physical modulation of microtubule dynamics, the post-translational modifications of tubulin including acetylation and polyglutamylation (Janke and Magiera, 2020) can adapt the growth dynamics of microtubules to regulate axonal branches. Particularly, tubulin acetylation controls axonal branching by regulating microtubule dynamics (Dan et al., 2018). Further, it is critical that the dynamic behavior of microtubules occurs under spatial and temporal coordination. The branch specific destabilization of microtubules by the enzyme spastin at neuromuscular synapses can actually mediate the loss of branches (Brill et al., 2016) instead of their induction. Altogether, these observations highlight a key role of microtubule dynamics in axon branching.

## Organelle Positioning at the Axonal Branch

While cytoskeleton re-organization unambiguously plays a crucial role in the structural support of axon branch formation, there are other cellular actions facilitating the process of branch formation and maintenance. Selective localization of cellular factors such as synaptic vesicles and mitochondria are observed (Courchet et al., 2013; Greif et al., 2013; Spillane et al., 2013; Tao et al., 2014; Figure 2A). In the next section, we will discuss the current understanding of the redistribution and potential role of these organelles at axon branch sites.

### Mitochondria

Mitochondria undergo bidirectional transport in axons, i.e., from the cell body to the synaptic terminal and vice versa; and their transport and redistribution along the axon is controlled by the coordinated action of the motor proteins kinesin and dynein, mitochondria adapter proteins such as TRAKs (Trafficking Kinesin Proteins)/Milton, and the anchoring protein, syntaphilin (Sheng and Cai, 2012). The perturbations of mitochondria-transport processes have been well studied in the context of neurodegenerative diseases, including Alzheimer’s disease, Huntington’s disease or amyotrophic lateral sclerosis (ALS) (Johri and Beal, 2012; Magiera et al., 2018; Guo et al., 2020). In healthy neurons, mitochondria are enriched in areas, which have high demands for energy production including synapses, growth cones and axon branch points (Sheng, 2017). The active transport of mitochondria into branching axons (Ruthel and Hollenbeck, 2003) suggests their role during branch formation. However, stalled mitochondria alone inside axons are not sufficient to induce a branch, as notably about 70% of mitochondria are stalled along axons at a given time (Cai et al., 2009). Nevertheless, evidence points toward the requirement of additional coordination of mitochondria by signaling and adapter proteins during branch formation. In cortical neurons, the depletion of liver kinase B1 (LKB1) or the anchoring protein syntaphilin leads to a decrease in the number of stalled mitochondria in axons and diminished axon branches, while the overexpression of these proteins leads to an increase of stalled mitochondria as well as associated axon branches (Courchet et al., 2013). Similar results are obtained by manipulating adenosine monophosphate-activated protein kinase AMPK signaling (Tao et al., 2014). The deletion of the mitochondria adaptor protein TRAK1 leads to a reduction of axon growth and branching (van Spronsen et al., 2013). Interestingly, studies have shown that fission and fusion events of mitochondria may control axon branching as well. For example, mitochondrial fission factor (MFF) has been shown to regulate the size and number of axonal mitochondria at presynaptic sites and show a correlation with axon branching (Lewis et al., 2018). Furthermore, neurotrophins, which promote axon branching, also induce the fission of mitochondria along sensory neurons (Armijo-Weingart et al., 2019). These studies suggest a role of mitochondria fission and the resulting increase in the number of mitochondria during branch formation.

### Endoplasmic Reticulum

The endoplasmic reticulum (ER) is a continuous network of sheets and tubes present in many cell types and organisms with diverse functions such as lipid synthesis, a platform for secretory protein synthesis, maintenance of calcium homeostasis and redistribution of membrane-associated proteins (Schwarz and Blower, 2016). In immature neurons before polarity formation, ER displays perinuclear cisternae and peripheral tubules that form a dense network (Nixon-Abell et al., 2016). In mature neurons, ER mostly shows a tubular morphology along the axon while it forms both tubular and planar network in dendrites (Wu et al., 2017). ER interacts with microtubules as well as different cellular organelles including mitochondria (Spillane et al., 2013; Wu et al., 2017). While ER has been implicated to play a role in establishing neuronal polarity and dendrite arborization (Renvoise and Blackstone, 2010), its role in axon branching is unknown. In less specialized mammalian cell lines such as Cos-7 and U2OS, ER has also been shown to mark the fission sites for mitochondria (Friedman et al., 2011) in an actin-dependent manner (Korobova et al., 2013). These observations raise the question whether there is an active role of ER in axonal branch formation by controling the fission process of mitochondria, to be addressed in the future.

It is not completely understood which ER-interacting proteins play a role in mediating the ER-mitochondria interactions in neurons. In HeLa cells, PDZD8 is present at ER-mitochondria contacts, and its deletion leads to decrease in the contact points (Hirabayashi et al., 2017). Within the axon shaft, ER protein p180 (also known as ribosome binding protein 1 homolog 180-kDa, RRBP1) interacts with both ER and microtubules, and contributes to the determination of the axon from neurites during the initial stage of the neuronal polarity formation (Farias et al., 2019). Furthermore, microtubules and ER stabilize each other to promote the growth of neurites. It is plausible that the co-migration of ER and microtubules occurs at the branch and is necessary for the stabilization of dynamic microtubules in a similar mechanism as the elongation of the axon shaft. Other ER-associated proteins that also interact with microtubules include CLIMP63, kinectin (KTN1) (Shibata et al., 2010), and atlastin-1. Particularly, atlastin-1 is enriched in vesicular structures at growth cones and branch points of cortical neurons in rats. Depletion of atlastin-1 results in compromised neuronal development (Zhu et al., 2006). Interestingly, atlastin-1 regulates the number of mitochondria at branching points in dendrites of sensory neurons (Liu et al., 2019), raising the possibility that atlastin-1 may have a similar function at axon branches. These studies strengthen the notion that ER is involved in the fission process of mitochondria. Together with mitochondria, ER and its interacting proteins appear to support the cytoskeleton and other organelles to promote axon branching.

Whether axons contain rough ER has been an open question. The presence of mRNAs coding for plasma membrane proteins and components of the secretory machinery gives the first indication of their existence and local translation in axons (Willis et al., 2007; Merianda et al., 2009). Conventional electron microscopic approaches show densities resembling rough ER at axonal tips (Jin et al., 2016). However, direct evidence for the presence of rough ER at the axonal branch is still lacking. Future studies will investigate how local translation is regulated at axon branching sites and growth cones, and how rough ER might participate in branching homeostasis.

## Membrane Remodeling at Axon Branches

### The Role of Synaptic Vesicles and Membrane Fusion in the Expansion of Axon Branches

Membrane extension at axonal branches and growth cones requires a plethora of proteins and lipid components (Figure 2B). They are mainly supplied by accumulated synaptic vesicles, dense-core vesicles, and ER membranes. Along the axon and branching sites, synaptic vesicles are reported to be present in high numbers (Winkle et al., 2016). These vesicles deliver membrane materials to the expanding plasma membrane by simple fusing or exocytosis via SNARE (soluble N-ethylmaleimide-sensitive factor-attached protein receptor) complexes (Sollner et al., 1993; Winkle and Gupton, 2016). While the fusion-mediated maintenance of membrane- and secretory materials at synapses of mature neurons is well known for signal transduction between neurons and neuromuscular junctions, it is not clear if such a fusion mechanism is utilized in axon development. Indeed, the presence of clustered synaptic vesicles and the accompanying exocytosis machinery has been shown in developing axons even before synaptogenesis (Kraszewski et al., 1995; Verderio et al., 1999). Moreover, the overexpression of these components in neurons enhances the number of branches (Perin et al., 1990; Alsina et al., 2001; Greif et al., 2013). Imaging of GFP-tagged synaptic vesicle components in neurons reveals insights into the potential function of these accumulated vesicles. Synaptobrevin II, a synaptic vesicle marker, is enriched at branching sites of retinal ganglion cells, and most of the new branches emerged from GFP-labeled sites (Alsina et al., 2001). When exposing neurons to brain-derived neurotrophic factor (BDNF), not only axon branching is increased, but also the density of GFP-synaptobrevin at branch points, showing the response of synaptic vesicles to extracellular stimuli. On the other hand, when the effective BDNF levels are reduced by neutralizing antibodies, a reduction in synaptobrevin levels and axonal branches occurs (Hu et al., 2005). These results together emphasize the importance of the accumulation of synaptic vesicles prior to axon branching as a resource for creating a new branching path. Importantly, these studies also point out that the use of synaptic vesicles can be dynamically adapted to the physiological needs during the development, maintenance and communication of neurons.

Similar to BDNF, Netrin-1, another extracellular signaling molecule, increases the number of axonal branches in cultured cortical neurons (Dent et al., 2004). A higher local concentration of Netrin-1 induces an increase in calcium transients in neurons (Hutchins and Kalil, 2008), possibly leading to the modulation of synaptic exocytosis and stimulated branch formation. Using cortical neurons, the involvement of synaptic vesicle fusion in increased branch formation has also been shown in response to Netrin-1 (Winkle et al., 2014). Terminal branches emerge from sites displaying high fluorescence intensity using fluorescently labeled synaptic-vesicle protein synaptophysin in live cell imaging in zebrafish and *Xenopus* retinotectal projections (Ruthazer et al., 2006). Notably, branches emerging from only faintly labeled puncta retract themselves, suggesting that the critical accumulation of synaptic vesicle components and their fusion with the plasma membrane (Winkle et al., 2014) is required for the maturation of axonal branches (Meyer and Smith, 2006; Ruthazer et al., 2006). Furthermore, the overexpression of syntaxin1-binding protein Sec1, which is involved in the formation of SNARE complexes (Jahn, 2000; Waters and Hughson, 2000), leads to a higher number of collateral axon branches in hippocampal neurons (Steiner et al., 2002). Altogether, these studies demonstrate that synaptic vesicles and their fusion with the plasma membrane is important for axonal branch formation.

### The Role of ER and Its Associated Proteins in the Expansion of Axon Branches

The ER in axons is in contact with the plasma membrane, which suggests that it provides necessary lipids for membrane expansion (Fowler et al., 2019; Figure 2B). ER and its associated proteins at axon branches contribute to membrane remodeling by regulating membrane fusion and the pool of synaptic vesicles. In *Drosophila* motor neurons, changing the levels of the ER-associated protein atlastin perturbs the release of synaptic vesicles along axons (De Gregorio et al., 2017). In contrast, overexpression of protrudin, an ER-resident protein, leads to membrane deformation and the formation of long neurites (Shirane and Nakayama, 2006), likely mediated by the interaction with the GDP-form of Rab11 and Kinesin-1. Both regulate the anterograde transport of recycling endosomes to the plasma membrane at growing axons (Shirane and Nakayama, 2006; Matsuzaki et al., 2011; Raiborg et al., 2015). A similar mechanism is found for protrudin in cultured cortical neurons as well as in injured optic nerves *in vivo* (Petrova et al., 2020). A recent study shows that protrudin interacts with ER via PDZD8, which has a lipid transferring activity at the contact sites of ER and endosomes (Shirane et al., 2020) and possibly at the ER-mitochondria interface (Hirabayashi et al., 2017). These point toward a role of protrudin as part of the lipid shuttling machinery to control the membrane expansion and shrinkage.

### Endocytosis for the Control of Membrane Retraction

Accumulating observations suggest that membrane expansion and retraction are controlled by membrane trafficking events. Considering the similarity of branching axons to growth cones, it is plausible that the expansion of membranes at branching axons is mediated by exocytosis, while the retraction of branched axons may be controlled by an intake of membrane through endocytosis (Figure 2B). The inhibition of endocytic pathways has been shown to increase the number of branches in different types of neurons (Zhou et al., 2007; Hausott et al., 2011; Kanamori et al., 2015), presumably due to the accumulation of branching signaling elements like NGFs and FGFR1. Furthermore, it has been shown that the membrane-curvature forming F-BAR (FER/CIP4 Homology Bin-Amphiphysin-RVS) family proteins, involved in endocytosis, negatively regulate axon branch formation (reviewed in Winkle et al., 2016). GFP-labeled Rab5 vesicles, that mark early endosomes, accumulate at axon branching sites (Ponomareva et al., 2014). This series of experiments shows that endocytic trafficking itself may regulate axon branching negatively, while exocytosis of synaptic vesicles correlates with the promotion of axon branching. In addition to these classical models of membrane retraction, Bishop et al. (2004) reported that axon branches at neuromuscular junctions are removed by a shedding of membrane-bound remnants, which contain mitochondria and synaptic vesicles. This particular mechanism could be advantageous for rapidly retracting axonal branches, especially for the axon pruning process. However, its regulation mechanism has yet to be shown.

## Concluding Remarks

Axon branching is a dynamic process that modulates the axonal architecture and fosters various cellular activities to ensure interconnections with neighboring neurons. It requires a highly coordinated and controlled organization of numerous cellular machineries. While many cellular processes at axon branches resemble those found in non-neuronal cells, some functions and regulatory features have been specifically adapted to axon branching. Despite a wealth of in-depth information on the axon branching process unveiled in recent years, many key questions are still open and part of active research: How are initial branching sites selected? What is the hierarchical sequence of molecular actions from the initiation of branching to a stabilized, mature branch site? How is the crosstalk of membranes and cytoskeleton components mediated? How are cellular components like synaptic vesicles and mitochondria shuttled to and stationed at branching sites? How is the assembly of the building blocks coordinated to achieve the formation of axon branches? Do all types of neurons share similar mechanisms, considering the diversity of different neurons and their morphologies? Answering these questions in the future will help to understand how neuronal circuit formation is facilitated on a molecular level.

## Author Contributions

SB and NM collected, analyzed the relevant literature, and wrote the review manuscript. HN prepared the figures with the help of the other authors. NB commented on and reviewed the manuscript prior to submission. All authors read and approved the submitted version.

## Conflict of Interest

The authors declare that the research was conducted in the absence of any commercial or financial relationships that could be construed as a potential conflict of interest.

## Publisher’s Note

All claims expressed in this article are solely those of the authors and do not necessarily represent those of their affiliated organizations, or those of the publisher, the editors and the reviewers. Any product that may be evaluated in this article, or claim that may be made by its manufacturer, is not guaranteed or endorsed by the publisher.
